# *Listeria* peritonitis in a patient on hemodialysis for end‐stage renal disease secondary to lupus nephritis—a case report

**DOI:** 10.1002/ccr3.7938

**Published:** 2023-09-19

**Authors:** Victor Frederick Cox, Alejandro Perez‐Albela, Michael Ramirez, Ryan Commins

**Affiliations:** ^1^ Georgetown University School of Medicine Washington DC USA; ^2^ Department of Internal Medicine MedStar Georgetown University Hospital Washington DC USA

**Keywords:** gastroenterology/hepatology, general medicine, infectious disease, nephrology, rheumatology

## Abstract

**Key Clinical Message:**

Patients with a recent history of peritoneal dialysis catheter removal in the setting of immunosuppression should be monitored for rare *Listeria* bacterial peritonitis. This infection should be managed with ampicillin and gentamicin.

**Abstract:**

There are scattered reports to date of patients with peritonitis caused by *Listeria monocytogenes*. *Listeria* peritonitis is more commonly reported in patients with cirrhosis and those receiving peritoneal dialysis. We present a case of *L. monocytogenes* peritonitis in a patient with end‐stage renal disease due to lupus nephritis actively on hemodialysis months after peritoneal dialysis catheter removal.

## 1 INTRODUCTION

*Listeria monocytogenes* is a gram‐positive, catalase‐positive, beta‐hemolytic facultative intracellular bacillus known to cause invasive disease in humans, particularly central nervous system (CNS) infection.^1^ Patients who are immunocompromised, pregnant, infants, old age, diabetic, or have alcohol use disorder have increased risk for infection with *Listeria monocytogenes*.^1^ *L. monocytogenes* is a rare cause of peritonitis and more commonly reported in patients with cirrhosis and those receiving peritoneal dialysis.^2^

## 2 CASE

We present a 46‐year‐old female with end‐stage renal disease due to systemic lupus nephritis currently on thrice weekly hemodialysis after recurrent episodes of peritoneal dialysis‐associated peritonitis requiring peritoneal catheter removal 10 weeks prior to presentation and untreated hepatitis C without cirrhosis who presented with a 1 day history of acute onset nausea, vomiting, watery diarrhea, and abdominal pain. She denied any headache, neck pain or stiffness, or photophobia. Her prior episodes of peritoneal dialysis‐associated peritonitis were due to *Enterococcus faecium* and *Candida parapsilosis*. Both were treated with antimicrobial therapy with resolution. The peritoneal dialysis catheter was removed after the second episode of peritonitis, and she was started on hemodialysis via a tunneled hemodialysis catheter in her left chest wall. Her last dialysis session was the day prior to presentation. The patient was on daily hydroxychloroquine 200 mg and prednisone 10 mg at home for systemic lupus erythematosus. On examination, her temperature was 37.3°C, pulse was 110, and blood pressure was 107/76, and she was visibly in pain with tenderness around the prior peritoneal dialysis catheter site. Blood work was significant for a leukocyte count of 14,760/mm^3^, with 87.8% neutrophils, and creatinine of 4.90. Liver function tests were unremarkable. *Clostridioides difficile* antigen testing was negative. CT of the abdomen and pelvis with IV contrast revealed moderate volume ascites with enhancement of the peritoneal fold. An lumbar puncture was deferred due low clinical suspicion of meningitis. A diagnostic paracentesis was performed with fluid analysis yielding a leukocyte count of 3873, with 89% neutrophils. Both ascites fluid and blood cultures grew *Listeria monocytogenes*. Prior to culture results, she was started on intravenous piperacillin–tazobactam 13.5 g daily, vancomycin 750 mg daily, and a 14‐day course of micafungin 200 mg daily and metronidazole 500 mg twice a day due to initial concern of recurrent *Candida* peritonitis. These antimicrobials were stopped after speciation and she completed an 18‐day course of ampicillin 2 g twice a day and gentamicin 2 mg/kg given post‐hemodialysis (three times a week). A paracentesis performed 1 day prior to completion of antibiotics revealed non‐neutrocytic and culture‐negative ascitic fluid.

## 3 DISCUSSION

*L. monocytogenes* is a gram‐positive bacillus known to cause zoonotic and invasive disease in humans. Listeriosis causes a spectrum of disease from a self‐limited febrile gastroenteritis to more invasive disease in susceptible patients with high mortality. Most cases are sporadic, but *Listeria* is a well‐recognized cause of foodborne outbreaks. *L. monocytogenes* is a ubiquitous organism that can be found in water, soil, animal feces, and vegetation. Human hosts are infected after ingestion of contaminated foods. *L. monocytogenes* then translocates from the gastrointestinal tract and disseminates from lymph nodes via the bloodstream to other organs. *Listeria* peritonitis is a rare presentation of listeriosis and more commonly occurs in patients with cirrhosis and those receiving peritoneal dialysis.^2^ Invasive listeriosis, such as bacteremia, CNS infection, maternal and neonatal infection, carries high mortality. In a recent large cohort, bacteremia and CNS infection carried a 45% and 30% 3‐month mortality, respectively.^3^ Accurate estimates for *Listeria* peritonitis‐related mortality are difficult to make due to its rare occurrence. However, only 21 cases of dialysis‐associated *Listeria* peritonitis have been reported to date including our patient, and of these patients, only 2 had a fatal outcome from their infections.^4^ Both these patients with a fatal outcome received initial antimicrobial treatment with vancomycin, highlighting the importance of early detection of *L. monocytogenes* in immunocompromised patients with peritonitis, in order to assure proper antibiotic coverage, which is comprised of ampicillin alone or ampicillin in combination with gentamicin.^5^, ^6^, ^7^ Similarly to other cases of *Listeria* peritonitis, our patient demonstrated resolution of *Listeria* infection after receiving ampicillin and gentamicin. This is the first case of *Listeria* peritonitis to be described in a patient status post peritoneal dialysis catheter removal thus not actively receiving peritoneal dialysis at the time of infection. For this reason, clinicians should be aware of and monitor for signs and symptoms for rare bacterial peritonitis even months after peritoneal dialysis catheters are removed as recent history of peritoneal dialysis appears to be a risk factor even in the setting of a patient who has transitioned to hemodialysis. In these patients, those who have symptoms of peritonitis such as nausea, vomiting, and abdominal tenderness should undergo diagnostic paracentesis with leukocyte count and culture of peritoneal fluid.^7^ Blood cultures should also be obtained to rule out bacteremia and systemic spread. Our patient's hospital course was also further complicated by recurrent ascites requiring multiple therapeutic paracenteses. There are several case reports that describe patients with systemic lupus erythematosus experiencing ascites concurrently with peritonitis, though a systematic review of gastrointestinal involvement in 2021 describes ascites as a function of protein losing enteropathy and one of the most frequent symptoms alongside diarrhea and generalized edema.^8^

In conclusion, patients either on peritoneal dialysis or recent history of peritoneal dialysis catheter removal should be monitored for peritonitis and rare bacterial peritonitis with organisms, such as *L. monocytogenes*, should be kept on the differential and detected early. Broad‐spectrum antibiotics are an ineffective treatment against *Listeria* peritonitis; therefore, infection should be managed with ampicillin and gentamicin.

## AUTHOR CONTRIBUTIONS

**Victor Frederick Cox:** Conceptualization; data curation; investigation; project administration; writing – original draft; writing – review and editing. **Alejandro Perez‐Albela:** Data curation; writing – original draft; writing – review and editing. **Michael Ramirez:** Data curation; writing – original draft; writing – review and editing. **Ryan Commins:** Data curation; supervision; writing – original draft; writing – review and editing.

## CONFLICT OF INTEREST STATEMENT

The authors have no conflicts of interests to declare.

## CONSENT

Written informed consent was obtained from the patient to publish this report in accordance with the journal's patient consent policy.

## Data Availability

Data available on request due to privacy restrictions.
